# Long-Term Outcomes of Phacoemulsification Surgeries at ECWA Eye Hospital: A Prospective Clinical Cohort Study

**DOI:** 10.1155/2024/2562064

**Published:** 2024-07-15

**Authors:** Mayor Orezime Atima, Ugbede Idakwo, Oyeronke Komolafe, Eisuke Shimizu, Nakayama Shintaro, Emmanuel Oluwadare Balogun, Emeka John Dingwoke, Ayodele Jacob Orugun, Olalekan Adebayo Ogundare, Pam Douglas Jah

**Affiliations:** ^1^ ECWA Eye Hospital, Kano, Kano State, Nigeria; ^2^ Department of Ophthalmology Keio University School of Medicine, Tokyo, Japan; ^3^ Department of Biochemistry Faculty of Life Sciences Ahmadu Bello University, Zaria, Kaduna State, Nigeria; ^4^ UNESCO-International Center for Biotechnology, Nsukka 410001, Enugu State, Nigeria

## Abstract

**Background:**

Phacoemulsification has proven to be a breakthrough technique in cataract surgery. Its popularity has grown dramatically as procedures and equipment have advanced, improving both safety and efficiency. This study presents long-term outcomes from phacoemulsification surgeries performed at the Evangelical Church Winning All (ECWA) Eye Hospital, a tertiary eye care center.

**Method:**

This prospective clinical cohort study followed standard practices for operations performed under local anesthesia. Ophthalmologists evaluated long-term outcomes and predictors of improved visual acuity after phacoemulsification cataract surgery. The visual recovery of the patients over time was evaluated, and the factors that influence the gains in vision were identified.

**Results:**

A total of 177 patients were subjected to treatment at our facilities during the study period. There were 116 male and 61 female patients, which resulted to a male-to-female ratio of 1 : 0.53. The average age of the patients was 59.18 years with a standard deviation of 11.38 years. Of the 259 eyes treated, 249 eyes (96.1%) achieved a high success rate with visual acuity of 6/6 – 6/18. Ten (10) eyes (3.9%) had moderate acuity between <6/18 and 6/60. Follow-up examinations over five years after phacoemulsification showed poor vision outcomes among old patients. The primary factor that affected improvement in visual acuity among patients was amblyopia, present in 30% of cases. Posterior capsular opacification and macular edema collectively accounted for 20% of poor vision cases, while optic atrophy, glaucoma, and retinal hemorrhage each represented approximately 10% of poor vision cases.

**Conclusions:**

The phacoemulsification approach demonstrated a highly effective restoration of vision for the vast majority, while long-term data analysis indicated the potential for age-related variability in postoperative visual gains.

## 1. Introduction

Cataracts remain the leading global cause of blindness, despite being readily treatable through conventional surgical procedures [1–3]. Visual impairment due to cataracts poses serious public health challenges through its social, economic, and psychological consequences [1]. By definition, cataracts describe any crystalline lens opacity, regardless of size, resulting from congenital, metabolic, traumatic, or age-related causes [4, 5]. This opacity scatters light and impairs vision. The only effective treatment is surgical removal of the clouded crystalline lens and implantation of an intraocular lens [6, 7]. Significant advances have improved cataract surgery, including innovations in anesthesia, phacoemulsification techniques, and intraocular lenses. Cataract surgery is now often performed outpatiently [2]. Phacoemulsification represents a modern extracapsular cataract extraction method. This approach emulsifies the lens using ultrasonic energy for eye aspiration, avoiding a large incision [5, 8]. Currently, phacoemulsification is the predominant surgical technique for the implantation of intraocular lenses. It is considered one of the safest and most effective procedures for its intended purpose [9, 10].

Cataract surgery techniques and results have advanced considerably in recent years. Smaller incisions are now a standard practice, and phacoemulsification is the preferred procedure for most surgeons [3]. Associated with these enhancements have been improvements in intraocular lens materials and designs, making them especially suitable for use through small incisions [11].

Before the development of phacoemulsification more than 20 years ago [12], surgeons faced significant challenges in the removal of cataracts and the implanting of intraocular lenses. Without phacoemulsification technology, surgeons had to remove the entire lens and capsule during extraction, making the placement of an intraocular lens difficult [3, 13]. The introduction of phacoemulsification represented a breakthrough in cataract surgery. Since its debut, the popularity of phacoemulsification has risen dramatically as the procedures and associated equipment have advanced. These improvements have improved both the safety and efficiency of cataract removal and intraocular lens implantation [10, 14]. This report presents the results of a long-term prospective clinical study of phacoemulsification cataract surgeries performed at the Evangelical Church Winning All (ECWA) specialty eye care center. By closely monitoring patients, the study hoped to gain insights that could guide expectations and help optimize care for future patients undergoing phacoemulsification.

## 2. Methods

### 2.1. Ethical Considerations

This study was approved by the Human Research Ethics Committee of the Eye Hospital of ECWA (ECWA/HREC/001/2017) and adhered to the ethical standards described in the Declaration of Helsinki, as amended in Edinburgh in 2000. The study design and conduct followed the guidelines established in STROCSS 2021 [15]. Results are reported according to the STROCSS criteria. Furthermore, the study was registered in the Research Registry [16], at https://www.researchregistry.com, with the Unique Identification Number of the Research Registry: 9767. Informed consent was obtained from all patients prior to the medical procedure and the use of their information, in accordance with ethical standards. Consent covered both surgical intervention and subsequent use of related medical records for research purposes.

### 2.2. Inclusion and Exclusion Criteria

This study included patients aged 18 years and older who provided informed consent and had operable cataracts without other ocular pathology. The patients were required to complete a minimum 5-year follow-up period. Exclusion criteria consisted of patients who did not provide consent, had ocular or systemic pathology unrelated to cataracts, did not complete the entire 5-year study duration, or were under 18 years of age. The aim was to evaluate the results over an extended follow-up period for patients undergoing cataract surgery without other complicating ocular health problems.

### 2.3. Surgical Procedures

This prospective cohort study was conducted at the ECWA Eye Hospital Kano from January 1 to December 31, 2017. Two hundred fifty-nine eyes were included in the study and followed for five years from 2018 to 2022 to evaluate the causes of poor vision. A peribulbar anesthesia technique was utilized, consisting of 3 ml of 2% xylocaine with 0.01% adrenaline. The periorbital area was prepped with 10% povidone iodine and draped. A lid speculum was inserted to expose the surgical field.

The ophthalmic surgeon created a clear corneal incision using a 3.2 mm keratome along with a paracentesis incision. After staining and washing the anterior lens capsule, a capsulorhexis procedure was performed under viscoelastic material. The nucleus was then emulsified and aspirated using an ultrasound phaco probe inserted through the main wound. The remaining cortical matter was removed from the capsular bag. The bag was filled with viscoelastic, and a foldable intraocular lens (IOL) was inserted through the main incision in place. An anterior chamber washout was conducted with a balanced salt solution. An intracameral antibiotic consisting of 0.1 ml of 5 mg/ml ceftriaxone was administered. This was followed by a subconjunctival injection of 2 mg of dexamethasone and 20 mg of gentamycin. A 0.3% drop in ciprofloxacin was also administered. For 24 hours after the operation, the eye was covered and shielded.

On the first postoperative day, the eye was examined. The evaluated parameters included corneal clarity, anterior chamber depth, anterior chamber flare, pupil size, IOL position, visual acuity, and intraocular pressure. Topical medications consisting of 0.05% dexamethasone qid, 1% mydriacyl daily, and 0.3% ciprofloxacin qid were prescribed for 4–6 weeks before tapering. Pain relievers were administered to patients experiencing pain. All patients with photophobia received sun shields.

### 2.4. Statistical Analysis

Data were analyzed using descriptive statistics in IBM SPSS version 19.0. Quantitative variables were summarized by their means and standard deviations in a 95% confidence interval. Categorical variables were expressed as proportions. A *p* value of less than 0.05 was used as the threshold for statistical significance.

## 3. Results

**Figure 1:**
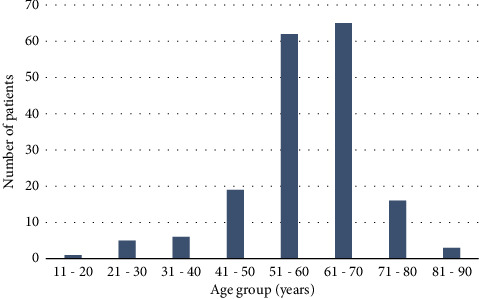
Age distribution of patients.

The patients recruited for this study comprised those who underwent phacoemulsification surgery within one year and were long-term followed for five years from January 2018 to December 2022. A total of 177 patients were treated in our facilities during the study period. There were 116 male and 61 female patients, representing a male-to-female ratio of approximately 1 : 0.53. Among the patients, those aged 11–20 years comprised the smallest group (0.6%), while those aged 61–70 years comprised the largest group, 36.7% (Figure 1). The average age range was 46 ± 11.35 years (Table 1). Of the 259 eyes studied (Table 2), the laterality was distributed as follows: the right eye in 50 patients, the left eye in 45 patients, and bilateral involvement in 82 patients.

Visual acuity outcomes after phacoemulsification surgery are presented in Table 2. Of the 259 eyes studied, 96.1% (249 eyes) achieved a visual acuity between 6/6 and 6/18, indicating a high success rate. The next largest group consisted of 10 eyes (3.9%) that had a moderate acuity record between <6/18 and 6/60. Poor vision was observed in 10 elderly patients during the 5-year postoperative follow-up period (Table 3).

**Figure 2:**
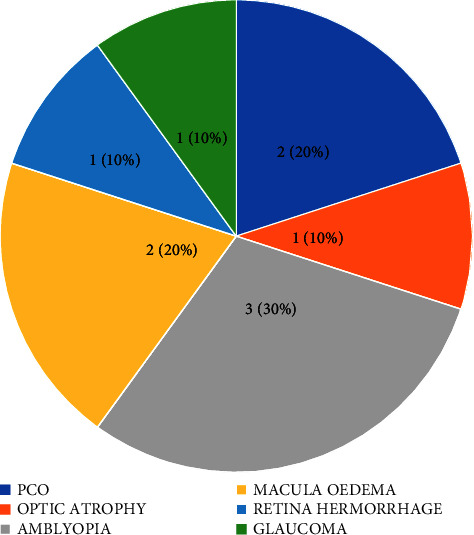
Causes of poor vision. PCO: posterior capsular opacification.

The distribution of poor vision observed during the five-year follow-up period and its causal factors are shown in Figure 2. Ten patients had poor vision during the 5-year postphacoemulsification follow-up period (Table 3). Amblyopia was the main cause of poor vision, accounting for 30% of the recorded cases. Posterior capsular opacification (PCO) and macular edema collectively represented 20% of cases, while optic atrophy, glaucoma, and retinal hemorrhage each represented 10% of poor vision cases. Poor vision was mainly observed in the left eyes, with six recorded cases, while the right eyes had four cases (Table 3).

## 4. Discussion

We present the long-term clinical results of patients who underwent phacoemulsification surgeries at the ECWA Specialist Eye Care Hospital in Kano State, Nigeria. The cohort consisted of more men than women, with a male-to-woman ratio of 1 : 0.53 and an average patient age of 59.18 ± 11.38 years (Table 1). Although much of the literature indicates that ocular diseases disproportionately affect women compared to men [17, 18], our findings were similar to a recent 10-year retrospective study of phacoemulsification surgeries [19], which reported a higher ratio of men (58.5%) than women (41.5%). Among the patients in the present study, those aged 51–60 years and 61–70 years dominated, representing 35% and 36.7% of the cohort, respectively (Table 1 and Figure 1). This is consistent with reports that worldwide, 65% of those with moderate-to-severe visual impairment or 82% blindness are 50 years and older [18, 20]. As in a previous study, [21] we did not find a clear predilection for laterality, as there were significant numbers of cases of the left eye, right eye, and bilateral.

Phacoemulsification remains the preferred surgical approach to cataract removal since its introduction by Charles Kelman in the 1960s [22]. In our medical facility, phacoemulsification cataract surgery was performed under local anesthesia and changes in visual acuity were recorded. In particular, 96.1% of the operated eyes achieved a good result with acuity of 6/6–6/18, while 3.9% experienced a moderate acuity change to <6/18−6/60, and there were no cases of poor outcome (Table 2). A related study reported an improvement in visual acuity of 96.4% in Malaysians after phacoemulsification surgery among cataract patients [23]. Given the very high rate of successful outcomes in our study, the data suggest that phacoemulsification is an appropriate technique for cataract surgeries. This is consistent with reports from similar studies that use phacoemulsification and recommend its use as a safe, rapid, and effective procedure [24–26].

During the course of our long-term follow-up of patients who underwent phacoemulsification surgery between January 1, 2018, and December 31, 2022, we found that ten patients exhibited poor vision outcomes (Table 3). Within this group of patients with poor vision, many were older individuals. Age could potentially explain the lower likelihood of significantly improved visual acuity after surgery in these cases. Previous research has shown that age is a significant prognostic factor affecting visual acuity gains after cataract surgery [23]. Specifically, older patients tend to have less robust visual improvement compared to younger cohorts. Additionally, studies have shown that patients with intraoperative complications or comorbid medical conditions have reduced chances of better vision after surgery [27]. Another published work found that those aged 80 years and older faced considerably higher risks of suboptimal or poor visual results after the procedure [28].

In this study, we evaluated the etiological factors that may have caused the poor vision observed in ten patients and estimated their relative percentages (Figure 2). This analysis helped us understand the degree to which each factor affected visual improvement after phacoemulsification surgery. Amblyopia was found to be the most significant factor affecting visual acuity improvement among patients after phacoemulsification surgery, comprising 30% (Figure 2). However, posterior capsular opacification and macular edema collectively constituted 20%, while optic atrophy, glaucoma (a set of disorders that damage the optic nerve of the eye, resulting in vision loss [21]), and retina hemorrhage each represented 10% of cases with poor vision. A previous study that assessed predictors of visual outcome after phacoemulsification cataract surgery reported that significant predictors that affect improvement in visual acuity included the presence of diabetic retinopathy, glaucoma, and high-risk surgical complications [29]. Furthermore, a study of the European Registry of Quality Outcomes for Cataract and Refractive Surgery found that ocular comorbidities such as macular degeneration, glaucoma, diabetic retinopathy, amblyopia, and others were the most important predictors of improvement in visual acuity [30]. Preoperative comorbidities were also found to predict poor visual acuity, including age-related macular degeneration, diabetes mellitus, and chronic pulmonary disease [31, 32]. Our center is a major provider of tertiary eye care in the populous northern region of Nigeria. This study represents the first report of long-term cohort visual outcomes and factors affecting visual acuity improvement after phacoemulsification surgery in this region.

The results demonstrate the efficacy and durability of phacoemulsification for cataract removal over time in a hospital setting. Using modern phacoemulsification methods, ophthalmologists at the ECWA eye hospital have helped many patients maintain or improve their vision in a minimally invasive and highly effective manner.

One limitation of this study is the inability to study modifiable and nonmodifiable risk factors that can influence the long-term prognosis after phacoemulsification surgery. Identifying such factors could provide valuable information to better set expectations and optimize patient care for future patients undergoing phacoemulsification. Future research that elucidates the impact of various risk parameters has the potential to advance knowledge and aid clinical decision-making for those undergoing this common ocular procedure.

## 5. Conclusions

The emergence of phacoemulsification as a technique for cataract surgery holds promise for potential benefits. It resulted in good visual acuity for 96.1% of cataract surgery patients. Long-term follow-up of postphacoemulsification outcomes suggested that older patients were less likely to experience significant visual improvement after the procedure. The findings also provide insights that can further optimize phacoemulsification and benefit even more people suffering from cataracts in the future.

## Figures and Tables

**Table 1:** Age distribution of the patient with phacoemulsification.

Age group	Frequency	Percentage (%)
11–20	1	0.6
21–30	5	2.8
31–40	6	3.4
41–50	19	10.7
51–60	62	34.8
61–70	65	36.7
71–80	16	8.9
81–90	3	1.7
Age (mean ± SD, years) 46 ± 11.35		

**Table 2:** Visual acuity of patients operated on.

Visual acuity	Remarks	Number of eyes	Percentage (%)
6/6–6/18	Good	249	96.1
<6/18−6/60	Moderate	10	3.9
<6/60–PL	Poor	0	0
Total		259	100

**Table 3:** Characteristics of poor vision seen 5 years postphacoemulsification follow-up.

S/no.	Gender	Age		Laterality		Visual acuity		Causes of poor vision
				RE	LE	RE	LE	
1	M	70	BE	RE	LE	6/18	6/24	Posterior capsular opacification (LE)
2	M	57	LE	Nil	LE	Nil	6/60	Optic atrophy (LE)
3	M	70	BE	RE	LE	6/36	6/12	Amblyopia (RE)
4	M	70	RE	RE	Nil	6/24	Nil	Macula edema (RE)
5	M	40	BE	RE	LE	6/12	6/24	Posterior capsular opacification (LE)
6	M	58	LE	Nil	LE	Nil	6/60	Macula edema (LE)
7	M	70	RE	RE	Nil	6/36	6/6	Retina hemorrhage (RE)
8	M	63	BE	RE	LE	6/60	6/6	Amblyopia (RE)
9	M	62	BE	RE	LE	6/5	6/60	Glaucoma (LE)
10	M	65	RE	RE	Nil	6/36	6/9	Amblyopia (LE)

## Data Availability

All data in the current study are available from the corresponding author (MOA) upon reasonable request.

## Abbreviations

ECWA: Evangelical Church Winning All
IOL: Intraocular lens
STROCSS: Strengthening the reporting of cohort studies in surgery.
